# Grid search approach to discriminate between old and recent inbreeding using phenotypic, pedigree and genomic information

**DOI:** 10.1186/s12864-021-07872-z

**Published:** 2021-07-13

**Authors:** Pattarapol Sumreddee, El Hamidi Hay, Sajjad Toghiani, Andrew Roberts, Samuel E. Aggrey, Romdhane Rekaya

**Affiliations:** 1 Department of Animal and Dairy Science, The University of Georgia, Athens, GA 30602 USA; 2 USDA Agricultural Research Service, Fort Keogh Livestock and Range Research Laboratory, Miles City, MT 59301 USA; 3 USDA Agricultural Research Service, Beltsville Agricultural Research Center, Beltsville, MD 20705 USA; 4 Department of Poultry Science, The University of Georgia, Athens, GA 30602 USA; 5 Institute of Bioinformatics, The University of Georgia, Athens, GA 30602 USA; 6 Department of Statistics, The University of Georgia, Athens, GA 30602 USA

**Keywords:** Ancient and recent inbreeding, Ancestral inbreeding, Beef cattle, Inbreeding depression, Purging, Runs of homozygosity

## Abstract

**Background:**

Although inbreeding caused by the mating of animals related through a recent common ancestor is expected to have more harmful effects on phenotypes than ancient inbreeding (old inbreeding), estimating these effects requires a clear definition of recent (new) and ancient (old) inbreeding. Several methods have been proposed to classify inbreeding using pedigree and genomic data. Unfortunately, these methods are largely based on heuristic criteria such as the number of generations from a common ancestor or length of runs of homozygosity (ROH) segments. To mitigate these deficiencies, this study aimed to develop a method to classify pedigree and genomic inbreeding into recent and ancient classes based on a grid search algorithm driven by the assumption that new inbreeding tends to have a more pronounced detrimental effect on traits. The proposed method was tested using a cattle population characterized by a deep pedigree.

**Results:**

Effects of recent and ancient inbreeding were assessed on four growth traits (birth, weaning and yearling weights and average daily gain). Thresholds to classify inbreeding into recent and ancient classes were trait-specific and varied across traits and sources of information. Using pedigree information, inbreeding generated in the last 10 to 11 generations was considered as recent. When genomic information (ROH) was used, thresholds ranged between four to seven generations, indicating, in part, the ability of ROH segments to characterize the harmful effects of inbreeding in shorter periods of time. Nevertheless, using the proposed classification method, the discrimination between new and old inbreeding was less robust when ROH segments were used compared to pedigree. Using several model comparison criteria, the proposed approach was generally better than existing methods. Recent inbreeding appeared to be more harmful across the growth traits analyzed. However, both new and old inbreeding were found to be associated with decreased yearling weight and average daily gain.

**Conclusions:**

The proposed method provided a more objective quantitative approach for the classification of inbreeding. The proposed method detected a clear divergence in the effects of old and recent inbreeding using pedigree data and it was superior to existing methods for all analyzed traits. Using ROH data, the discrimination between old and recent inbreeding was less clear and the proposed method was superior to existing approaches for two out of the four analyzed traits. Deleterious effects of recent inbreeding were detected sooner (fewer generations) using genomic information than pedigree. Difference in the results using genomic and pedigree information could be due to the dissimilarity in the number of generations to a common ancestor. Additionally, the uncertainty associated with the identification of ROH segments and associated inbreeding could have an effect on the results. Potential biases in the estimation of inbreeding effects may occur when new and old inbreeding are discriminated based on arbitrary thresholds. To minimize the impact of inbreeding, mating designs should take the different inbreeding origins into consideration.

**Supplementary Information:**

The online version contains supplementary material available at 10.1186/s12864-021-07872-z.

## Background

The negative impact of inbreeding on complex traits (i.e., the reduction in mean phenotypic values due to inbreeding), known as inbreeding depression, is likely due to increased homozygosity of loci carrying partially recessive deleterious alleles (partial dominance hypothesis) [1]. These unfavorable alleles are maintained at low frequency via mutation-selection balance [2]. However, involvement of some loci with heterozygote advantage, maintained at intermediate frequencies by balancing selection, can also lead to inbreeding depression (overdominance hypothesis), although its role is less evident [3]. Individual level of inbreeding (F) is an estimate of the probability of identity by descent (IBD) of alleles at a locus due to common ancestral origin [4, 5]. Inbreeding depression is a measure of the effects of inbreeding on traits. Traditionally, individual inbreeding was estimated based on pedigree information (F_ped_), and is, thus, a measure of the expected proportion of the genome that is autozygous (homozygous due to the inheritance of IBD alleles) [4]. As expected, the pedigree-based measure of inbreeding is highly influenced by the quality of the pedigree (accuracy) and its completeness [6, 7]. Simulation and real data results have shown an underestimation of true inbreeding using incomplete or inaccurate pedigrees [8].

With the availability of high-density single nucleotide polymorphisms (SNPs), several genomic estimators have been proposed to assess inbreeding. These genomic estimators measure the realized autozygosity and are independent of the depth and completeness of the pedigree. Several studies showed superiority of using genomic data to estimate true inbreeding compared to F_ped_ [8, 9]. Inbreeding calculated based on stretches of homozygous SNP marker genotypes, known as runs of homozygosity (ROH), is one of the best genomic estimators. ROH segments arise when two identical haplotypes are inherited from a common ancestor, thus, they are mainly autozygous genome segments [10]. Since ROH segments are less likely to arise by chance, inbreeding coefficients calculated based on ROH (F_ROH_) [11] tend to be more accurate in estimating the realized autozygosity, and it has been shown to be a powerful method to assess the effects of inbreeding [12]. Inbreeding depression is predominantly caused by rare and recessive variants, and F_ROH_ is a better predictor of homozygosity at rare variants [13]. ROH segments are enriched for deleterious recessive alleles [14–17], supporting the ample evidence of association between ROH segments and inbreeding depression in livestock [18–22]. Furthermore, the distribution of ROH segment length could be a valuable resource to distinguish between recent and ancient inbreeding as ROH length correlates with the distance (number of generations) to a common ancestor [19, 21, 23, 24]. Long ROH segments are likely produced by recent common ancestors (recent inbreeding) due to the limited time for recombination to break up long stretches of autozygosity, whereas shorter segments are likely to have been generated longer ago, reflecting older inbreeding [25, 26]. In fact, the age of inbreeding, measured by the number of generations (*g*) to a common ancestor, can be inferred from the expected length of ROH segments that follows an exponential distribution with mean equal to $\frac{1}{2g}$ Morgan [27]. This information is useful for determining the impact of inbreeding and to better understand the purging of deleterious alleles [28]. In addition, assessment of the risk posed by short and long ROH segments requires knowledge of the extent to which ROH segments of different lengths contribute to inbreeding depression [15].

The level of inbreeding depression is expected to vary across populations [29]. Populations may purge deleterious recessive alleles over generations when undergoing inbreeding, thus limiting the degree of inbreeding depression [30]. Purging decreases the frequencies of deleterious recessive alleles over time under artificial or natural selection [31]. Thus, similar levels of autozygosity could have different effects on traits depending on their age. Consequently, ancient inbreeding (inbreeding arising from distant common ancestors) is expected to have less harmful effects than recent inbreeding (inbreeding rising from more recent common ancestors). Obviously, the effectiveness of purging in removing the harmful effects of inbreeding depends on several factors such as the rate of accumulation of autozygosity, the level of selection pressure, the effect sizes of deleterious alleles, the environmental conditions, and the purging process (nonrandom mating or genetic drift), among others [31–35]. Studies of purging are largely based on the concept of ancestral inbreeding [36, 37], which measures the cumulative proportion of alleles within a genome that have undergone inbreeding in the past and are therefore exposed to natural selection. In livestock populations, ancestral inbreeding was found to be associated with purging of inbreeding depression [21, 38, 39]. Alternative approaches that explicitly examine harmful impacts of new and old inbreeding on a trait are based on quantifying the contribution of recent ancestral generations in the calculation of new inbreeding [21, 40–43]. These approaches estimate inbreeding by tracing the pedigree back to a pre-specified number of generations. The latter will be used as a threshold to identify recent inbreeding. Unfortunately, this threshold is arbitrarily set, and it is very likely to be population or even breed dependent. Several studies have recently attempted to discriminate between recent and ancient inbreeding using genomic autozygous segments [21, 23, 44, 45]. Several approaches have been proposed to categorize ROH segment length into different age classes based on arbitrary thresholds [21, 23], model-based clustering procedures [46, 47], and hidden Markov models to model homozygous by descent [48]. These various approaches (pedigree or ROH-based) used to discriminate between recent and old inbreeding have led to inconsistent results with regard to the effects of inbreeding on various traits, suggesting the lack of a well-defined approach to classify inbreeding. Consequently, depending on definitions of recent and old inbreeding, their effects on phenotypes vary greatly and may deviate from the expectation that new inbreeding is more harmful.

Time plays an important role in allowing selection to purge deleterious recessive mutations, therefore minimizing their impact compared to inbreeding that originated from more recent common ancestors. This assumption that time alters inbreeding effects could be used to better classify inbreeding into recent and old age classes. The ability to identify inbreeding classes associated with a greater impact on phenotypes (inbreeding depression) could be useful not only for quantifying its impact on phenotypes, but also for better herd management to minimize inbreeding depression more efficiently. For example, age distribution of inbreeding (e.g., recent inbreeding derived from long ROH segments) in selection candidates can be used to optimize mating schemes, especially in small populations or some breeding programs where consanguineous mating is inevitable. The Line 1 Hereford cattle herd is a valuable population for the characterization of inbreeding due to the availability of a well-recorded and deep pedigree together with moderately dense SNP data [49]. The objectives of this study are to: 1) develop a new method to distinguish between recent and old inbreeding using pedigree and ROH information and 2) compare the performance of the proposed approach with existing methods for growth traits using the Line 1 Hereford cattle population.

## Results

### Phenotype and pedigree data

A basic summary description of the phenotypic data used in this study is presented in Table 1. Analyzed traits consisted of birth weight (BW), weaning weight (WW), yearling weight (YW), and average daily gain between weaning and yearling (ADG). Only animals with both genotypic and phenotypic information were used.

**Table 1:** Summary description of phenotypic data for genotyped animals

Trait/covariate^**a**^	n	Mean	SD	Min	Max
BW, kg	743	37.30	4.64	21.77	53.52
WW, kg	736	197.68	34.12	96.62	293.02
YW, kg	687	338.14	81.30	169.64	555.65
ADG, kg/d	687	0.844	0.352	0.150	1.625
Weaning age, d	736	180.8	15.9	131	215
Yearling age, d	687	345.1	18.1	286	403

^a^*BW* birth weight, *WW* weaning weight, *YW* yearling weight, *ADG* average daily gain, *Weaning age* age at collecting WW, *Yearling age* age at collecting YW

**Fig. 1:**
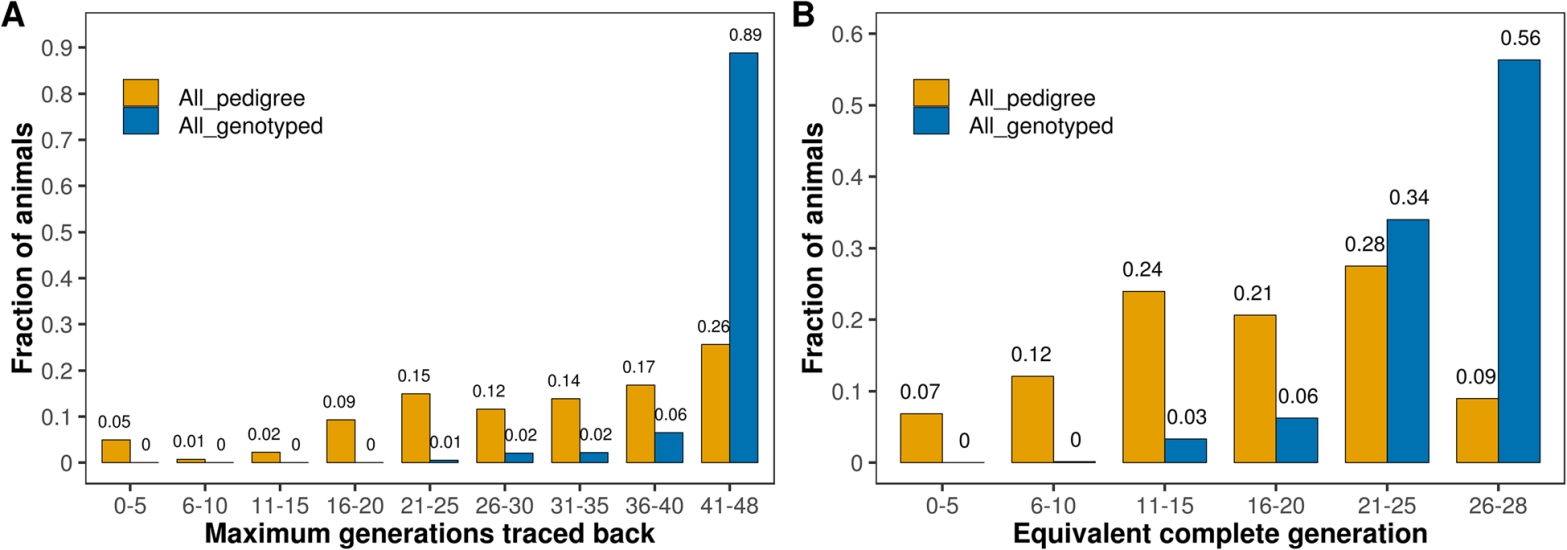
Distribution of (**A**) maximum number of traced back generations (MaxGen) and (**B**) equivalent complete generation (ECG) for all (All_pedigree) and only genotyped (All_genotyped) animals

The Line 1 Hereford cattle population is a unique resource to dissect inbreeding due to its long-term linebreeding and its deep and relatively complete pedigree information [49]. Almost all (> 99%) and around 89% of genotyped animals had more than 20 and 40 ancestral generations tracing back to their earliest ancestors, respectively (Fig. 1A; Table 2). As expected, the mean of F_ped_ increased with increase in number of generations traced (MaxGen) and ranged between 0.03 and 28.73% when MaxGen was less than five and 48 generations, respectively (Table 2). However, for the majority of the MaxGen levels, several animals had missing pedigree inbreeding due to missing one or both parents (Fig. S1). Otherwise, more than 90% of the genotyped animals had more than 20 equivalent complete generations (ECG) (Fig. 1B; Table S1). The depth and completeness of the pedigree of Line 1 Hereford population provided a unique resource for the comprehensive dissection of the age of inbreeding and assessment of its effects on inbreeding depression.

**Table 2:** Distributions of pedigree based inbreeding (F_ped_, % ) for different maximum number of generations to the earliest ancestor

Maximum generations^**a**^	All animals (***n*** = 10,478)				Genotyped animals (***n*** = 785)			
	n	Mean (SD)	Min	Max	n	Mean (SD)	Min	Max
≤ 5	516	0.03 (0.71)	0	16.02	0	–	–	–
6–10	72	3.64 (6.22)	0	22.34	0	–	–	–
11–15	235	3.14 (4.75)	0	23.04	0	–	–	–
16–20	973	12.33 (7.80)	0	36.01	0	–	–	–
21–25	1566	21.74 (5.44)	10.62	42.19	4	23.25 (3.00)	20.40	26.68
26–30	1216	24.41 (3.01)	16.68	43.38	16	24.11 (1.58)	21.42	26.70
31–35	1454	26.89 (3.98)	0	46.35	17	26.81 (2.21)	22.59	31.86
36–40	1765	24.60 (10.58)	0	46.74	51	28.44 (1.48)	25.98	31.38
41–48	2681	28.73 (6.51)	0	39.98	697	29.50 (5.45)	0.00	39.98

^a^Number of generations between an animal and its earliest ancestor (MaxGen)

### Discrimination between old and recent inbreeding based on pedigree and ROH information

**Fig. 2:**
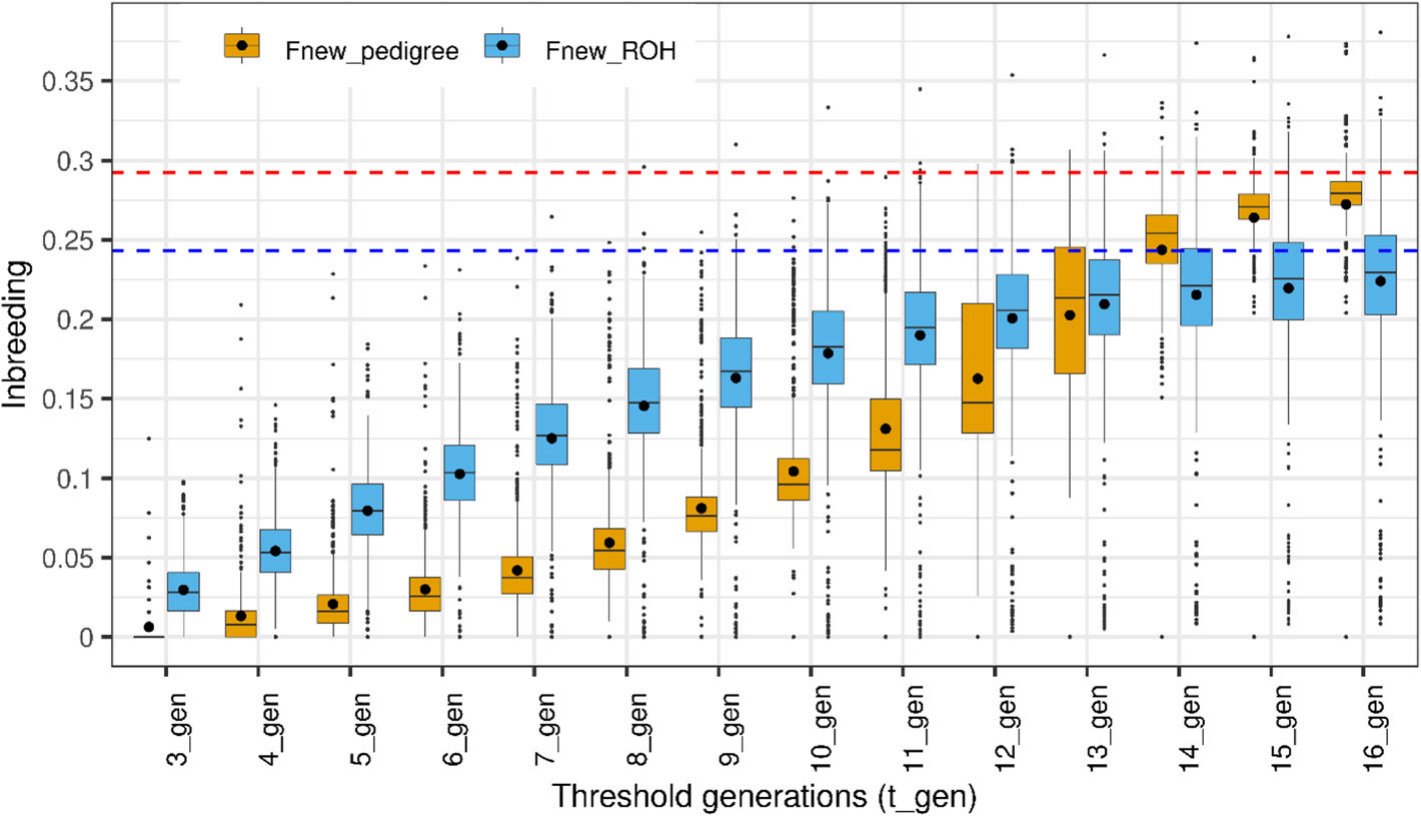
Recent inbreeding as a function of the number of generations threshold (t _ gen) used to define new inbreeding based on pedigree (Fnew_pedigree) and ROH segments (Fnew_ROH). Total inbreeding based on pedigree (**F**_**ped**_) and ROH segments (**F**_**ROH**_) are represented by the red and blue horizontal lines, respectively

Figure 2 presents the change in recent inbreeding (without standardization) as a function of the number of generations using pedigree and genomic information. As described in the method section, inbreeding was dissected into recent (new) and ancient (old) classes using a changing base generation approach and subjective length thresholds for pedigree and ROH segments, respectively. The relationships of an individual back to a specified threshold generation (*t* generations) were traced using pedigree information. When using ROH information, segments were clustered into short (old) and long (new) classes based on pre-defined length thresholds (*m* Mb). Recent inbreeding was defined as all inbreeding that occurred up to the threshold generation *t*, and all other inbreeding was considered ancient or old inbreeding. Figure 2 illustrates that ROH segments captured a greater amount of new inbreeding compared to the pedigree at the same number of generations, particularly when the threshold is less than 13 generations. When new inbreeding was defined up to nine generations, the rate of increase in inbreeding was clearly greater for new ROH inbreeding. When the threshold is greater than 13 generations, the pedigree new inbreeding increases at a faster rate compared to its ROH counterpart, which seems to have reached a plateau.

**Fig. 3:**
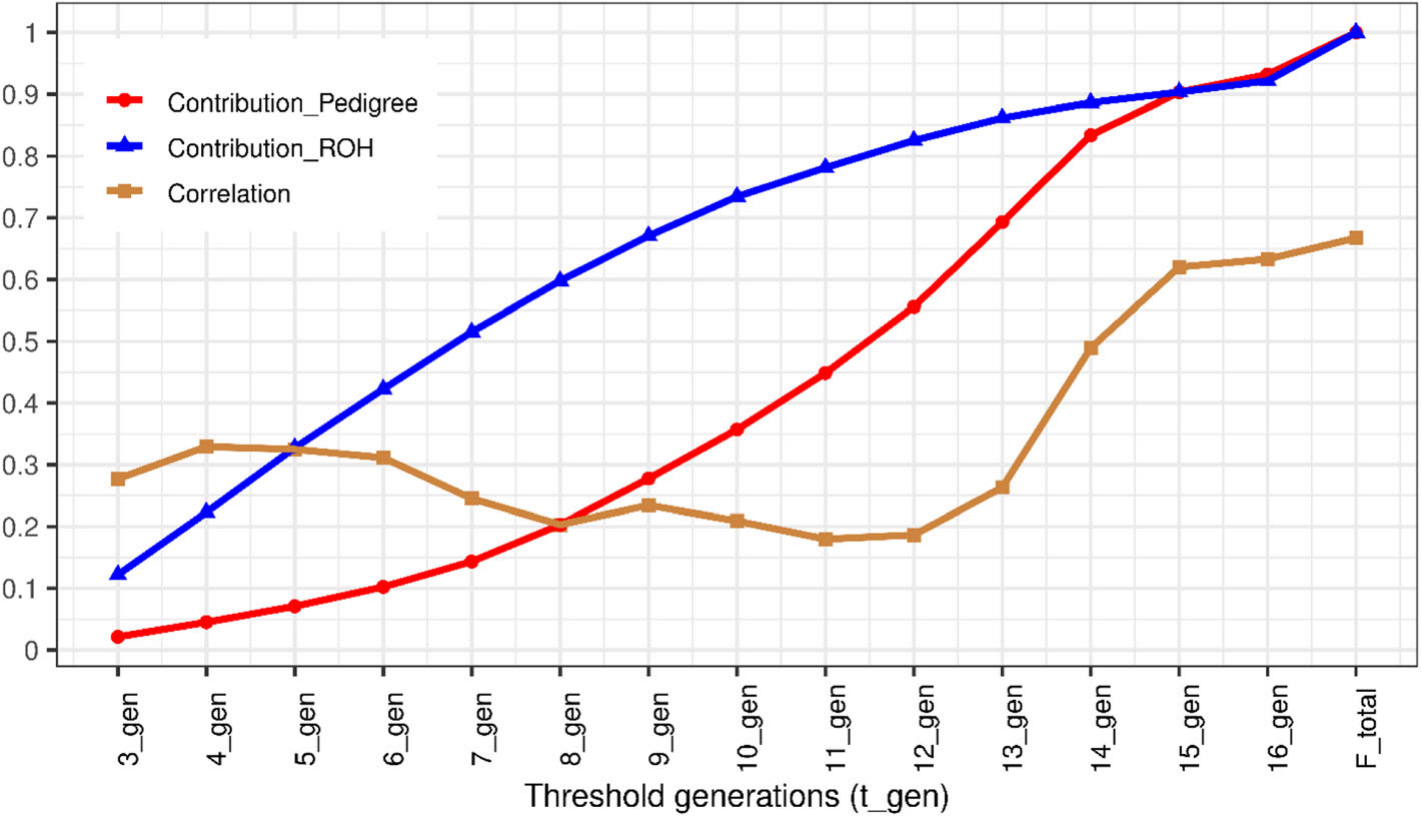
Contribution of recent inbreeding based on pedigree (Contribution_Pedigree) and ROH (Contribution_ROH) to the total inbreeding and their correlation as a function of the number of generations threshold (t _ gen) used to define new inbreeding

Figure 3 shows that the relative contribution of new inbreeding to total inbreeding is significantly higher when using ROH segments compared to using pedigree at the same number of generations, particularly when the threshold generation is less than or equal to 13. For instance, new inbreeding accounted for 50% of the total inbreeding at seven and 12 generations using ROH and pedigree information, respectively. Contribution of new inbreeding to total inbreeding was similar for both sources at 13 or more generations (Fig. 2), and it reached about 90% of the total inbreeding when the threshold was set to 15 generations (Fig. 3). In other words, if new inbreeding is defined based on 15 ancestral generations for pedigree or ROH segments longer than 3.3 Mb (assuming 100 Mb per 1 Morgan), it will have the same contribution to the total inbreeding. The correlation between new inbreeding coefficients calculated based on pedigree and ROH segments was low and ranged between 0.18 and 0.32 for the first three to 13 generations (Fig. 3). After 15 generations, the correlation increased to approach the correlation between total pedigree and ROH inbreeding coefficients (0.667).

Thresholds to discriminate between new and old inbreeding were determined separately for each trait and thus they are trait specific. As indicated before, the basic assumption is that recent inbreeding is more detrimental compared to its ancient counterpart. In order to facilitate the interpretation of their relative contributions, recent and old inbreeding were standardized to have a zero mean and a variance of one (Z-scores) separately in each inbreeding depression analysis. By assessing a series of different potential cut-off thresholds, the threshold value that results in a detrimental effect of new inbreeding exceeding that of its old counterpart will be declared as the classification threshold.

**Fig. 4:**
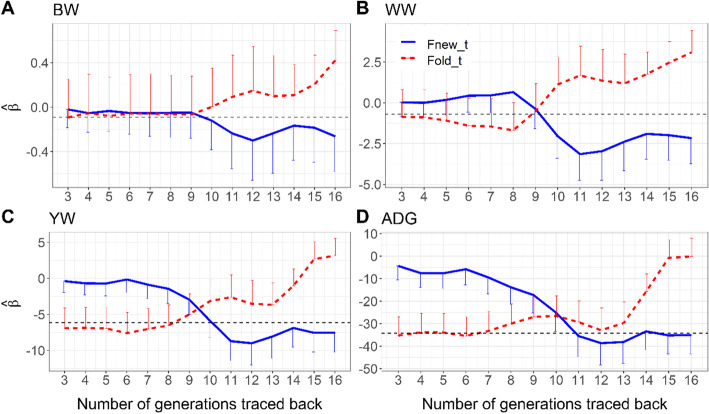
Estimates of the regression coefficients associated with new (**F**_**new** _ **t**_**)** and old (**F**_**old** _ **t**_) pedigree based inbreeding as a function of the number of generations used to discriminate between new and old inbreeding for birth (BW, kg) (**A**), weaning (WW, kg) (**B**), yearling weights (YW, kg) (**C**) and ADG (gram/day) (**D**). The horizontal lines indicate the inbreeding depression estimates based on total inbreeding. Error bars indicate standard errors

**Fig. 5:**
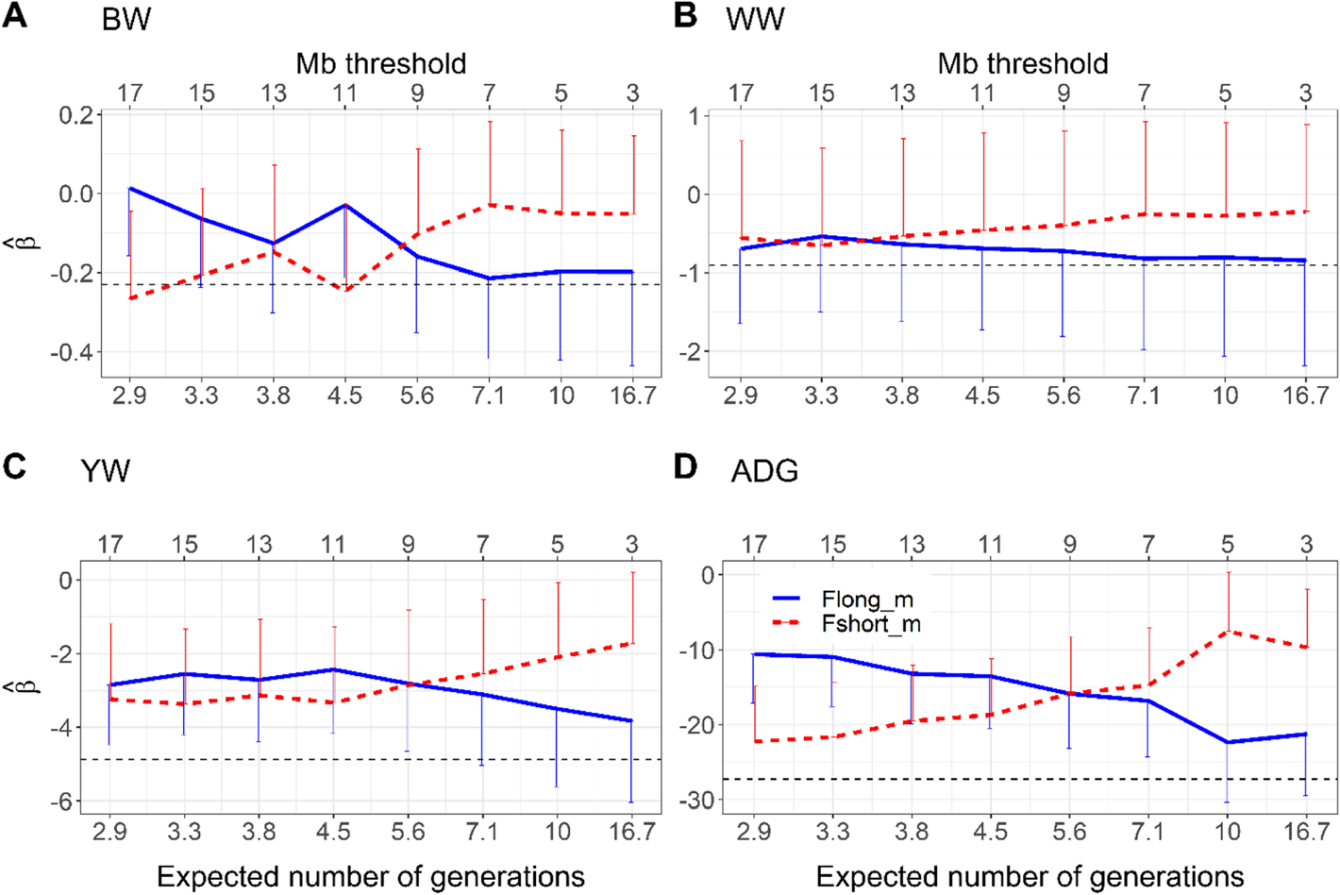
Estimates of the regression coefficients associated long (**F**_**long** _ **m**_) and short (**F**_**short** _ **m**_) ROH segments as a function of the threshold (in Mb) used to discriminate between new (long) and old (short) inbreeding for birth (BW, kg) (**A**), weaning (WW, kg) (**B**), yearling weights (YW, kg) (**C**) and ADG (gram/day) (**D**). The horizontal line indicates the inbreeding depression estimates based on total inbreeding. Error bars indicate standard errors. On the x-axis, the ROH segment’s length thresholds (Mb threshold) are presented on the top and their corresponding expected number of generations to common ancestors are presented on the bottom

The effects of new and old inbreeding on the four growth traits evaluated using pedigree- and ROH-based approaches, are shown in Figs. 4 and 5, respectively. When the pedigree-based inbreeding was partitioned based on the number of generations (*t*) back to a common ancestor, there was a clear divergent pattern in the direction of the regression coefficients associated with new and old inbreeding (Fig. 4). With exception of BW, when the number of generations used to define the threshold was small, the derived new inbreeding had a less harmful impact on the growth phenotypes compared to its old counterpart. As the threshold increased, the negative impact of newer inbreeding became more noticeable and it overcame the effect of its older counterpart. The point at which the change of pattern occurs is the threshold for new and old inbreeding classification. Using our approach, inbreeding arising from common ancestors 10 generations back is considered as new (recent) for BW, WW, and YW. For ADG the threshold is around 11 generations (Fig. 4).

Patterns of regression coefficients for new and old inbreeding effects on the four traits estimated using ROH information followed similar trends as observed using pedigree information; however, divergence between the effects of new and old inbreeding was less evident (Fig. 5). The length threshold (*m*) for new inbreeding (F_long _ m_) was 9 Mb for BW, 13 Mb for WW and 7 Mb for YW and ADG corresponding roughly to six, four, and seven discrete generations to common ancestors, respectively. At the evaluated cut-off generation thresholds (Fig. 3), the contribution of new inbreeding based on pedigree and ROH segments to the total inbreeding was less than 50%, except for ADG using ROH segments. At these estimated thresholds, new inbreeding accounts for at least as much as its old counterpart in inbreeding depression, yet its contribution to total inbreeding is less than 50%. This suggests that regardless of sources of information and trait, new inbreeding is more likely to account for the largest portion of the deleterious impact of inbreeding. Changes in the magnitude of estimated effects of long and short ROH-based inbreeding on a trait is influenced by the choice of the range of the cut-off point length threshold (*m*). In the current study, the chosen range was between 3 and 17 Mb. Shorter length thresholds were not considered due to the limitations of the density of the SNP panel to accurately identify very short ROH segments. The upper bound for the threshold (17 Mb) was chosen to reflect common ancestors going back three generations to mimic the minimum generation threshold used in the pedigree-based approach using the $\frac{1}{2g}$ relationship between segment length and number of generation [27, 50] ($\frac{1}{2\mathrm{x}17\ \mathrm{Mb}}\approx 2.94\ \mathrm{generations}$, assuming 1 Morgan = 100 Mb). It was observed that increase in threshold length led to a drastic increase in number of animals without long ROH segment class (about 15% of animals had no ROH segments longer than 19 Mb compared to less than 8% when the cut-off point was set at 17 Mb). Therefore, our predefined threshold settings were chosen to ensure sufficient information on different length ROH segments. This limitation could have some effects on the estimates of inbreeding and inbreeding depression.

Across all thresholds based on number of generation (*t*) or segment length (*m*), old and new inbreeding had no significant effects on BW and WW (Figs. 4 and 5). New inbreeding at *m* = 7 Mb was significantly associated with inbreeding depression for ADG ($\left|\frac{\hat{\upbeta}}{\mathrm{SE}}\right|>2$), while both new and old inbreeding at 11 generation threshold showed significant effects on ADG. However, the signal was not consistent for YW where only new inbreeding at 10 generation-threshold significantly caused a reduction in the trait. It should be noted that when using ROH segments to classify inbreeding into new and old classes, both short and long ROH classes had a negative impact on all growth traits for almost all predefined *m* thresholds (Fig. 5). In fact, estimates of inbreeding depression due to total inbreeding were significant only for YW and ADG ($\left|\frac{\hat{\upbeta}}{\mathrm{SE}}\right|>2$) irrespective of the source of information (pedigree or ROH) as indicated in Figs. 4 and 5.

### Comparisons between different inbreeding classification methods

A descriptive summary of the estimates of new and old pedigree-based inbreeding using existing and the proposed method is presented in Table S2. Existing methods consisted of using an arbitrary generation threshold (five generations) and the ancestral inbreeding approach following Kalinowski et al. [37]. Using existing methods, new inbreeding has limited contribution to total inbreeding (7.1 to 19.1%). Using the proposed method, new inbreeding accounted for 35.7 to 44.9% to the total inbreeding. It is worth mentioning that the cut-off thresholds were higher for the proposed method (10 and 11 generations). When ROH segments were used (Table S3), the contribution of new inbreeding (long ROH segments) to total inbreeding increased substantially for the existing methods compared to the situation when the pedigree was used. For the proposed method, the contribution of new inbreeding to total inbreeding decreased as expected with the increase of the cutoff threshold. There has been variation in the estimates of inbreeding coefficients and their associated standard deviations (SD) across methods and sources of information. However, variation in the standard deviations associated with age-specific inbreeding (new and old) were small and ranged between 1 to 5% and 1 to 4% using pedigree and ROH information, respectively (Table S2 and S3). Summary descriptions of the distribution of short and long ROH segments using different approaches can be found in Table S4.

Model comparisons of the proposed approach with existing methods using pedigree and ROH information are presented in Tables 3, respectively. Inbreeding depression models included covariates of new and old inbreeding and the systematic effects specific to each trait (see Methods section). Across the different comparison criteria (Adj.R2, RMSE, AIC, and BIC), the proposed approach performed similar to or better than existing methods. In general, the proposed approach performed better using pedigree for all traits compared to ROH information. Using pedigree (Table 3), the proposed method was better than existing approaches for all traits, with the majority of the differences being statistically significant (AIC difference > 2). Although differences were not statistically significant when ROH information was used, the proposed method was marginally better for BW and ADG, but was slightly inferior for WW and YW when compared to existing methods (Table 3). Overall, classification of inbreeding based on the proposed approach will lead to an adequate fit to observed data (as indicated by Adj.R2 and RMSE), and a better prediction of future records (as indicated by AIC and BIC), particularly when applied using pedigree information.

**Table 3:** Model comparisons between the proposed and existing methods to classify inbreeding into new and old classes using pedigree and genomic (ROH segments) information

Trait^**a**^	Inbreeding^**b**^		Criterion^**c**^			
			Adj.R2	RMSE	AIC	BIC
***Using Pedigree information***						
BW	Existing	Kalinowski	0.1561	4.1822	4294.78	4433.10
		P_5L	0.1553	4.1841	4295.45	4433.78
	Proposed	P_10P	**0.1580**	**4.1776**	**4293.12**	**4431.44**
WW	Existing	Kalinowski	0.5418	22.6225	6741.76	6884.40
		P_5L	0.5402	22.6625	6744.36	6887.00
	Proposed	P_10P	**0.5436**	**22.5781**	**6738.87**	**6881.51**
YW	Existing	Kalinowski	0.8448	31.3207	6744.06	6884.56
		P_5L	0.8441	31.3937	6747.26	6887.76
	Proposed	P_10P	**0.8449**	**31.3088**	**6743.54**	**6884.04**
ADG	Existing	Kalinowski	0.8427	0.1366	− 726.03	− 590.06
		P_5L	0.8437	0.1361	− 730.21	− 594.24
	Proposed	P_11P	**0.8441**	**0.1359**	**− 732.16**	**− 596.19**
***Using Genomic information***						
BW	Existing	ROH_Mclust	0.15583	4.1829	4295.01	4433.34
		ROH_5L	0.15566	4.1833	4295.16	4433.48
	Proposed	ROH_9P	**0.15588**	**4.1828**	**4294.97**	**4433.29**
WW	Existing	ROH_Mclust	**0.53964**	**22.6759**	**6745.23**	**6887.87**
		ROH_5L	0.53946	22.6802	6745.52	6888.15
	Proposed	ROH_13P	0.53962	22.6762	6745.25	6887.89
YW	Existing	ROH_Mclust	0.84299	31.5019	6751.99	6892.49
		ROH_5L	**0.84304**	**31.4976**	**6751.80**	**6892.30**
	Proposed	ROH_7P	0.84299	31.5025	6752.01	6892.52
ADG	Existing	ROH_Mclust	0.84202	0.1369	−723.05	− 587.08
		ROH_5L	0.84205	0.1368	−723.15	−587.18
	Proposed	ROH_7P	**0.84211**	**0.1368**	**−723.43**	**−587.46**

^a^*BW* birth weight (kg), *WW* weaning weight (kg), *YW* yearling weight (kg), *ADG* average daily gain (kg/d)

^b^*Kalinowski* classification of inbreeding based on Kalinowski et al. (2000), *P_5L* classification of inbreeding based on 5 generations according to literature, *P_10P and P_11P* classification of inbreeding based on 10 and 11 generations, respectively, as determined by the proposed method, *ROH_Mclust* classification of inbreeding based on model-based clustering method, *ROH_5L* classification of inbreeding based on 5 Mb according to literature, *ROH_7P ROH_9P, and ROH_13P* classification of inbreeding based on 7, 9, and 13 Mb, respectively, as determined by the proposed method

^c^*Adj.R2* Adjusted R-squared, *RMSE* Root Mean Squared Error, *AIC* Akaike’s Information Criteria, *BIC* Bayesian information criteria

## Discussion

Inbreeding and inbreeding depression have been extensively studied in human and animal populations due to their influence on genetic diversity, viability, conservation, health, and productivity. However, the mechanism underlying inbreeding depression is still not fully understood. Homozygosity is not expected to have the same deleterious effects on traits independently of its age. Recent inbreeding arising from more recent common ancestors is likely to have greater negative impacts than older inbreeding because selection has had less time to remove deleterious alleles from the population. In the current study, pedigree- and ROH-based inbreeding were decomposed into new and old components guided by their effects on growth traits. The main tool of the proposed method to dissect inbreeding was the assumption that recent inbreeding is more harmful than old inbreeding.

As presented in Figs. 2 and 3, classification of inbreeding into new and old classes could be achieved using information contained in the pedigree and ROH segments. The thresholds to discriminate between new and old inbreeding based on our approach were trait specific. Using two sources of information (pedigree and ROH segments), we were able to discriminate between new and old inbreeding through the assessment of their corresponding effects on four growth traits (Figs. 4 and 5). When pedigree information was used, thresholds separating new and old inbreeding were clearly identified. In contrast, when ROH segments were used, the discrimination between new and old inbreeding was less evident. This could be due to the limited density of the marker panel, the small number of genotyped animals, the uncertainty in the identification of ROH segments, purging efficiency, as well as the variability in effects of ROH segments. In general, using medium-density SNP panels, long ROH segments are identified with high reliability as they are likely to be IBD. However, short segments have a higher probability of being non-IBD [51, 52]. For the bovine 50 K SNP marker panel, a minimum length of ROH segments of 4 Mb was advised by Ferenčaković et al. [52] while a limit of 5 Mb was suggested by Purfield et al. [51]. In the current study, the minimum length threshold to identify ROH segments was set to 1 Mb. Although such a requirement provides the possibility of tracking ancient inbreeding, it increases the risk of false detection of short autozygous segments. Additionally, the efficiency of purging of deleterious alleles from short ROH segments depends on several factors. In fact, Sumreddee et al. [53] showed using the same data set and minimum length threshold (1 Mb) that the efficiency of purging depended on selection pressure and rate of inbreeding within the population. Thus, short ROH segments may still harbor harmful alleles in spite of their distant origin. Furthermore, the large number of short ROH segments, each potentially with small effect, could collectively have a non-negligible contribution to inbreeding depression [53]. All these factors combined, complicate the discrimination between ROH segment classes using the proposed approach (Fig. 5 and Table 3). This is expected as the proposed method relies on comparing the magnitudes of unfavorable effects of recent and old inbreeding.

Using pedigree information, our proposed approach resulted in a significantly better fit compared to existing methods for all traits (AIC difference > 2) (Table 3). However, there was no significant difference between methods using ROH segments; although the proposed method had slightly better fit for BW and ADG (Table 3). In general, the quality of fit of the model (AIC and BIC) using pedigree information seems to be slightly better than when ROH segments were used. This might be due to the high quality (depth and completeness) of the pedigree information and the potential noise in the identification of ROH segments. The discrepancy between the different model comparison criteria (Tables 3) is not unexpected due to their statistical properties. While AIC and BIC account for model complexity (effective number of parameters in the model) on top of the quality of fit (minimizing of the sum of square of the residuals), R2 accounts only for the quality of fit.

It should be noted that when our approach is compared with the method based on the concept of Kalinowski’s ancestral and new (F_anc _ Kal_ and F_new _ Kal_) inbreeding using pedigree information (Table 3), the results should be interpreted with caution. In this study, the version of Grain program used to compute F_anc _ Kal_ and F_new _ Kal_ could lead to biased estimates of inbreeding coefficients compared to the new version of the software as demonstrated by Doekes et al. [54]. However, the differences in the estimates of inbreeding coefficients based on the two versions of the software are too small to have any meaningful impact on the results.

Across traits, lower thresholds for discriminating between new and old inbreeding were identified when ROH segment information was used compared to pedigree (3–7 compared to 10–11 generations), as indicated in Figs. 4 and 5. These results may indicate the ability of ROH segments to capture the deleterious effects of recent homozygosity much faster than the pedigree information. However, the smaller thresholds (in number of generations) obtained using the ROH segments could be influenced by the assumed relationship between the genetic (cM) and the physical distance (Mb) and the potentially large variance of the mean segment length (1/2 g) due to the likely low recombination rate in longer ROH tracts. Furthermore, at the same number of generation thresholds to classify inbreeding, new inbreeding had a higher contribution to the total inbreeding using ROH segments than pedigree information (Figs. 2 and 3). In spite of the relatively complete and deep pedigree of Line 1 Hereford population [49], recent inbreeding estimated based on pedigree may not capture all the realized fractions of IBD alleles transmitted over generations due to randomness during meiosis (i.e., pedigree-based inbreeding reflects the expectation of true inbreeding [55]). In contrast, ROH-based metrics could better reflect the realized proportion of the genome that is autozygous and thus better capture actual deleterious mutations [11, 12].

Thresholds to discriminate between short and long ROH segments are generally heuristically defined to reflect the age of IBD segments in most previous literatures such as dogs [15], pigs [23, 56], goats [57], sheep [58, 59] and cattle [16, 21, 41, 45, 60–62]. An alternative method based on modeling the distribution of ROH sizes (model-based clustering algorithm) has revealed a variation in the boundary thresholds across human populations [14, 46, 47, 63]. Although these population-specific thresholds are more informative to infer parental relatedness rather than using an arbitrarily predefined threshold applied equally in all populations [64], our proposed method will further expand the specificity of these thresholds to be trait-dependent. This will be useful particularly when the emphasis is on limiting deleterious effects of inbreeding is more urgent for a particular trait. When compared with the existing two-component mixture Gaussian clustering model (Mclust) implemented in mclust (v.5) R package [65] described in the methods section, our proposed method identified similar thresholds (e.g., 9.03 vs 9 Mb for BW).

Despite the extensive literature on pedigree-based inbreeding in livestock species, only a handful of studies have previously focused on the effects of new and old inbreeding. Their consistent conclusion was that inbreeding arising in recent generations had more harmful impacts than that from more distant generations [21, 42, 44, 66]. This is consistent with the expectation that frequencies of deleterious alleles are likely to decrease over time due to selection and purging, which influence the magnitude of inbreeding depression. Nevertheless, not all studied traits showed a clear difference between the effects of new and old inbreeding. In the pedigree-based study in mice by Hinrichs et al. [42], a sequence of thresholds (number of recent generations) was used to divide inbreeding into new and old components, and inbreeding depression was estimated for the size of the first litter. Without confounded effects of inbreeding rate (as a constant rate of inbreeding), they concluded that new inbreeding caused greater depression than old inbreeding when at least 25 generations (out of over 125 generations) were considered for new inbreeding. It should be noted that the number of generations that determined new inbreeding is not informative for comparisons across studies primarily due to different mating strategies, pedigree depths, and generation intervals. Interestingly, the contribution of new inbreeding to the total inbreeding was consistent between theirs and the present study using our proposed approach. The new inbreeding based on 25 generations [42] and on 10 generations from the present study (for BW, WW, and YW) contributed approximately 35% to the total inbreeding, and this fraction may serve as an indicator of the most recent generations for classifying new inbreeding. However, this agreement should be interpreted with caution due to different populations and traits analyzed. In fact, the method introduced by Hinrichs et al. [42] was investigated during the preliminary study and a relatively strong correlation (*r* > 0.99, *P* < 0.00001) was observed between their estimates of old and new inbreeding and their counterparts obtained using the changing base generation approaches used in the current study. Similar results were reported for litter size for pigs by Rodríguez et al. [66] using the same procedure to decompose inbreeding as presented in Hinrichs et al. [42]. Doekes et al. [21] reported a more harmful impact of inbreeding derived from recent generations compared to more distant generations on yield traits in dairy cattle, but no clear pattern was observed for fertility and udder health traits. The same pedigree-generation-based approach was used by Makanjuola et al. [44]. They concluded that inbreeding generated in the most recent five generations had significant unfavorable effects on milk production and fertility traits in Canadian Holstein cattle, whereas inbreeding from more remote generations was not significant. Studies on recent inbreeding depression using five pedigree generations as the threshold showed varying results in pigs [40] and cattle [41]. Silió et al. [40] studied the effects of pedigree inbreeding obtained from the five most recent generations compared with complete pedigree (26 generations) on post-weaning growth traits in pigs. They found that both partial and complete inbreeding had similar negative effects on daily growth rate and weight at 90 days of age, indicating the great influence of recent inbreeding. A study of semen quality in Austrian Fleckvieh cattle showed a significant effect of complete pedigree inbreeding (F_ped_) on the total number of spermatozoa, but not for recent inbreeding based on a five generation threshold [41]. Given the above, some of the variation in inbreeding depression across studies could be in part explained by the effectiveness of the purging process. Inbreeding should rapidly purge deleterious alleles from a population if a major portion of inbreeding depression is due to rare and highly deleterious recessive mutations present in homozygous form [2]. In other words, if the partial dominance hypothesis is a major cause of inbreeding depression, purging is more likely to remove harmful alleles over time within a population. However, the effectiveness of purging is limited for loci exhibiting overdominance [67]. Additional factors such as the rate of increase in level of inbreeding (slow rate of inbreeding promotes purging), genetic architecture of the trait (e.g., purging is more effective for large effect alleles), selection pressure (high selection pressure promotes purging), and population size (purging is more likely in small than large population) could affect the effectiveness of purging [1, 21, 30, 31, 33, 68, 69].

Length of ROH segments provides an excellent alternative to characterize the age of haplotypes and inbreeding. Long ROH segments are likely due to recent inbreeding, while short ROH segments reflect distant inbreeding [70]. Older haplotypes (short ROH segments) are more likely to carry small effect deleterious mutations that continue to segregate over a long period of time since large negative effects tend to be removed by selection (purging of disadvantageous alleles) in few generations [33]. On the other hand, long ROH segments are produced from new haplotypes and are likely associated with more recent inbreeding [11, 12, 46]. Several studies have found that the predicted deleterious variations in humans were enriched in long ROH compared to shorter ROH segments [14, 63]. In contrast, a study in cattle by Zhang et al. [16] reported the significant enrichment of predicted deleterious variants in short (< 0.1 Mb) and medium (0.1 to 3 Mb) ROH regions compared to longer regions (> 3 Mb). Using known deleterious recessive mutations in domestic dogs, Sams and Boyko [15] recently reported that the enrichment of these mutations was similar across different ROH length categories. Enrichment of deleterious recessive mutations in ROH regions is believed to be the cause of inbreeding depression. Although the extent to which ROH segments harbor deleterious alleles varies across species, enrichment of short and long ROH segments with known harmful mutations in dogs [15] supports our findings for some of the analyzed traits.

Previous studies showed that the effects of different ROH classes varied across traits and populations [13, 21, 23, 44, 45, 47]. Wang et al. [47] reported the associations between long ROH class and an increased risk of lung cancer in Han Chinese, whereas a decreased risk was associated with shorter ROH class. Saura et al. [23] classified ROH into short and long ROH classes based on a 5 Mb threshold, and found no inbreeding depression from these ROH classes on litter size in a highly inbred strain of pigs. However, they identified inbreeding depression at the chromosome level for recent inbreeding (long ROH) and were able to locate genes with deleterious effects. Similar approaches were used to classify ROH segments into five length classes to assess the effects of recent and more distant inbreeding in dairy cattle [21, 44]. Doekes et al. [21] reported a mixed contribution of short and long ROH segments to inbreeding depression on yield, fertility and udder health traits in Holstein-Friesian dairy cows. These results are consistent with the findings of this study. Mixed results were also reported in a large meta-analysis study of 100 human complex traits by Clark et al. [13], where the effects of short (< 5 Mb) and long ROH (> 5 Mb) ROH segments were examined. Makanjuola et al. [44] concluded that inbreeding due to long ROH classes (recent inbreeding) had more pronounced unfavorable effects compared to shorter ROH segments for milk production and fertility traits in Holstein dairy cattle. Maltecca et al. [45] recently used an arbitrary number of generations from one to four and four to eight generations ago to investigate the effects of new and old inbreeding based on a homozygous by descent (HBD) approach using 67,905 SNPs. Using yield deviations from Holstein cows, they reported a greater effect of recent inbreeding for all traits compared to old inbreeding. Understandably, comparison of results obtained from different studies is difficult. The variation in the results between these studies could be attributed partly to the approaches used to identify ROH segments (e.g., sliding window and model-based methods), number of genotyped animals, and differences in SNP densities. The latter dictates how well short segments (old inbreeding) and their associated effects are captured. In addition, the inconsistent results between the effects of long and short ROH classes across traits and thresholds could be partially explained by the distribution of ROH segments and inbreeding throughout the genome as reported in a previous study using the same data set [49].

In this study, inbreeding depression was significant for YW and ADG based on total (F_ped_ and F_ROH_) and age-specific inbreeding (new and old) despite the small sample size. Using pedigree and ROH segment information, new and old inbreeding had no significant effect on BW and WW. This could be due to unintended natural or artificial selection, causing highly inbred calves to die early before the phenotypes were recorded. In addition, preferential collection of data mainly from “live and non-abnormal calves” will likely affect the estimation of inbreeding effects especially on early growth performance and BW [49]. Birth weight is a trait subjected to stabilizing selection usually without favoring deleterious or beneficial alleles unless frequencies are high [39]. Intermediate phenotypes for birth weight are desirable and genotypes pushing birth weight outside the optimum middle range are disadvantageous. Apart from natural selection (and adaptation), the results are expected in part due to artificial selection to improve calving ease in the population in recent years [71]. Additionally, the small sample size and the potential purging of harmful alleles may explain some of the insignificant effects of inbreeding on BW and WW observed in this study. Variation in inbreeding depression due to age-specific inbreeding (new and old) may affect the power of the association test. The power to detect inbreeding depression depends on the sample size and the variance of inbreeding [12]. Although the latter may affect the results of this study, its impact is likely to be small due to the limited variation in SD of estimated age-specific inbreeding across methods and source of information (Table S2 and S3).

It should be noted that the proposed method to discriminate between the rising age of inbreeding relies heavily on the partial dominance hypothesis of inbreeding depression for which there is strong support [2, 31]. If inbreeding depression is caused by decreased frequency of beneficial heterozygotes (overdominance hypothesis) due to inbreeding, the effectiveness of purging will depend on the degree to which the loss of heterozygotes affects fitness [72]. Thus, inbreeding depression can result from old and new inbreeding. This may have some effects on the estimation of thresholds for discriminating between new and old inbreeding using our approach.

## Conclusions

Over time, selection tends to reduce or even eliminate deleterious alleles. Therefore, more recent inbreeding is likely to have a greater contribution to inbreeding depression. Distinguishing between new and old inbreeding could provide a better assessment of genome-wide inbreeding and facilitate management of the harmful effects of inbreeding depression largely caused by accumulation of deleterious mutations. A new method to classify inbreeding into recent and ancient classes was proposed and successfully applied to the inbred Line 1 Hereford population. Pedigree and ROH segment-based approaches were developed to decompose inbreeding based on the assumption that new inbreeding is more harmful than its old counterpart. The boundary thresholds for distinguishing new and old inbreeding appear to be trait specific. Pedigree and ROH information seemed to have different ability in classifying inbreeding into new and old classes. Pedigree information resulted in a clearer distinction between classes. However, use of ROH segments resulted in smaller thresholds in generations needed to detect new inbreeding. Such a discrepancy may be due to high variation in the distribution of ROH segments throughout the genome. Compared to existing approaches to classify inbreeding, our proposed method using ROH segments provided similar performance that was slightly better than using pedigree information on a population with extensive pedigree data. In populations without known pedigree information, the capacity to obtain genomic information could provide for inbreeding assessment. Short and long ROH segments appear to have an effect on the analyzed traits, supporting the hypothesis that inbreeding depression is caused by the accumulation of effects of deleterious alleles. Potential biases in the estimation of inbreeding effects may occur when the thresholds for new and old inbreeding are arbitrarily set. To minimize the impact of inbreeding, mating designs should avoid the pairing not only of closely related individuals but also of those with more recent parental relatedness, particularly in a small population. The proposed method will be very useful in designing mating schemes that minimize the impact of inbreeding depression on the most important traits by using animals with the largest old inbreeding component based on these trait specific thresholds. Future studies should focus on identifying regions of recent (long ROH) and old (short ROH) inbreeding that are associated with a deleterious/favorable impact on phenotypes to better understand the genetic basis of inbreeding depression.

## Methods

### Data: animals, genotypes, and phenotypes

Data used in this study were previously compiled, and no animal was euthanized or released during this study. The data originated from purebred Line 1 Hereford cattle maintained in a small closed herd at USDA-ARS, Fort Keogh Livestock and Range Research Laboratory, Miles City, MT [71, 73, 74] and has been previously used for investigating inbreeding and its impacts using pedigree and genotypic information [49]. Briefly, the Line 1 Hereford herd was founded in 1934 based on two paternal half-sib males and 50 unrelated females and was maintained under a careful mating scheme to minimize inbreeding [71, 75]. Management specific to this herd has been previously described [71, 76, 77]. Historically, the objective of selection in Line 1 was focused on increasing yearling weight [75]. Since 2011, the selection has been primarily focused on improving calving ease (direct and maternal) while maintaining weaning and yearling weights to at least herd averages.

A complete pedigree (recorded up to 2016) comprising 10,478 animals was used in the calculation of inbreeding coefficients. Pedigree analysis of the Line 1 population showed relatively complete, deep genealogical records [49] with an average of 17.15 and 25.36 equivalent complete generations (ECG) for all and only genotyped animals, respectively (Fig. 1B and Table S1). ECG is computed as the sum of all ${\left(\frac{1}{2}\right)}^{\mathrm{n}}$ terms, where n is the number of generations separating an individual from each of its ancestors and is used as a measure of pedigree quality (i.e., pedigree depth and completeness) that indicates the distance to the reference population where all individuals are unrelated.

A subset of 797 animals born between 1953 and 2016 was genotyped with different density SNP panels (3 k to 50 k markers). Informative imputed genotypes for 30,220 SNPs from 29 autosomal chromosomes for 785 animals (473 males, 312 females) were used in the analysis. Imputation accuracy ranged between 94 and 96.5% [78]. A detailed description of the imputation process and quality control, using the same data set, could be found in Sumreddee et al. [49]. Average distance between adjacent SNPs was 80.51 Kb with a standard deviation of 5.83 Kb. Growth data (BW = birth weight; WW = weaning weight; YW = yearling weight; and ADG = average daily gain post weaning) of animals born between 1990 and 2016 with both parents known were used (Table 1). For a reliable comparison of inbreeding depression using pedigree- and genomic-based approaches, only animals with more than 10 ECG were used.

### Grid search approach to discriminate between old and recent inbreeding

To determine the appropriate threshold for discriminating between new and old inbreeding, we developed a grid search algorithm based on the basic assumption that recent autozygosity has more harmful effects than old inbreeding. Thus, the threshold separating old and recent inbreeding can be identified simply by comparing their effects on trait phenotypes. By varying the threshold to cluster ROH into short and long segment classes, the optimal cut-off point to discriminate between recent and ancient inbreeding can be inferred. Since inbreeding coefficient’s variance varies with the threshold value, a standardization (*z*-transformation) was applied to make the regression coefficients associated with inbreeding (inbreeding depression) comparable when determining the thresholds for the discrimination between old and new inbreeding [79, 80]. However, this standardization will not have an effect on the estimation of inbreeding depression or biological interpretation of the results.

Standardized coefficients for new and old inbreeding based on varying thresholds (ROH length or number of generations) were included as covariates in the model for analyzing inbreeding depression. The magnitude of the estimated inbreeding effects (regression coefficients) associated with the partitioned inbreeding were then compared, and a cut-off point threshold for recent inbreeding was determined. The threshold was defined as the minimum number of ancestral generations (*t*) or the longest length *m* (in Mb) used to classify ROH segments that resulted in a larger negative effect of recent inbreeding compared to its old counterpart. The resulting new and old inbreeding are denoted hereafter as F_new _ t_ and F_old _ t_ using pedigree information and F_long _ m_ and F_short _ m_ using ROH segments. Obviously, the threshold is trait specific.

In order to evaluate the validity of the proposed method, results were compared to several existing approaches used to partition inbreeding into new and old classes.

#### Changing base generation approach

This pedigree-based approach simply traces the relationships of an individual back to a specified number (*t*) of ancestral generations (*t* = 3 to 16 in the current study), creating different subsets of the pedigree each with its base generation. The inbreeding coefficient (F_ped _ t_) for an individual is computed based on its partial pedigree information. When *t* is set at the maximum value, an individual will be traced to its earliest ancestor in the pedigree (MaxGen) and F_ped _ t_ will be equal to the total inbreeding (F_ped_) computed using all pedigree information. Therefore, the inbreeding level of an individual can be partitioned into two components:

1 $$ {\mathrm{F}}_{\mathrm{ped}}={\mathrm{F}}_{\mathrm{new}\_\mathrm{t}}+{\mathrm{F}}_{\mathrm{old}\_\mathrm{t}}, $$

where F_new _ t_ = F_ped _ t_ and F_old _ t_ = F_ped_ − F_new _ t_. The INBUPGF90 program [81] was used to calculate inbreeding coefficients for each individual using different values for *t*.

#### Subjective thresholds based on Runs of homozygosity segments

The expected length of a DNA segment that is IBD decays with the number of generations (*g*) since it arises from a common ancestor due to recombination events, following an exponential distribution with mean equals $\frac{1}{2g}$ Morgan [27]. Assuming a uniform rate of recombination (across the genomes and sexes), for simplicity, a mean genetic distance of 1 centiMorgan (cM) per 1 Mb [82] was used to derive the age of ROH length (i.e., age of inbreeding). Using this approach, ROH segments were clustered into short and long classes based on subjective thresholds. For a given threshold *m* (m = 3, 5, 7, 9, 11, 13, 15, or 17 Mb), the genome-wide inbreeding coefficient for an individual (F_ROH_) is decomposed into short (old) and long (new) components:

2 $$ {\mathrm{F}}_{\mathrm{ROH}}=\frac{\sum {\mathrm{L}}_{\mathrm{ROH}}}{{\mathrm{L}}_{\mathrm{AUTO}}}={\mathrm{F}}_{\mathrm{short}\_\mathrm{m}}+{\mathrm{F}}_{\mathrm{long}\_\mathrm{m}}, $$

3 $$ {\mathrm{F}}_{\mathrm{short}\_\mathrm{m}}=\frac{\sum {\mathrm{L}}_{\mathrm{ROH}\_\mathrm{short}}}{{\mathrm{L}}_{\mathrm{AUTO}}}, $$

4 $$ {\mathrm{F}}_{\mathrm{long}\_\mathrm{m}}=\frac{\sum {\mathrm{L}}_{\mathrm{ROH}\_\mathrm{long}}}{{\mathrm{L}}_{\mathrm{AUTO}}}, $$

where L_ROH_ and L_AUTO_ are the total length of all ROH segments and the autosomal genome covered by SNP (in Mb), respectively. F_short _ m_ and F_long _ m_ are the inbreeding coefficients due to short and long ROH segments based on threshold length value *m* (Mb) that were calculated with the total length of all short (L_ROH _ short_) and long (L_ROH _ long_) ROH segments, respectively.

Identification of ROH segments was performed using a sliding window approach as implemented in PLINK version 1.9 [83, 84]. Parameter settings for ROH identification in this population were previously described by Sumreddee et al. [49]. Briefly, the *--homozyg* function was used to perform ROH analysis. A maximal gap between two SNPs of 500 kb and a minimal density of 500 kb/SNP were used to increase the ROH genome coverage. No linkage disequilibrium pruning was performed prior to the analysis as it can lead to a biased ROH detection. These settings were recommended for medium SNP density panels to improve the quality of ROH analysis and the accuracy of inbreeding (F_ROH_) estimates [85]. In order to minimize the impact of potential genotyping errors, we allowed a maximum of two heterozygous SNPs within the sliding windows. A segment was declared as an ROH if it satisfied a minimum length threshold of 1 Mb and it harbored a minimum of 15 SNPs. The length threshold for ROH calls was chosen due to the limited information on short segments with the current density of the marker panel (Table S5). ROH-based inbreeding coefficients were estimated using in-house developed R scripts [86]. Example source code for the estimation of ROH-based inbreeding is provided in Appendix 1.

#### Ancestral inbreeding approach

his purging-based approach splits the classical inbreeding into ancestral inbreeding component (F_anc _ Kal_), representing homozygous alleles that have already been IBD in the past, and a new inbreeding component (F_new _ Kal_) reflective of alleles being IBD for the first time, as proposed by Kalinowski et al. [37]. Kalinowski ancestral pedigree inbreeding was calculated using a gene dropping approach with 10^6^ replications as implemented in PEDIG software [87]. Note that F_new _ Kal_ is different from new inbreeding (F_new _ t_) defined in eq. (1) using the changing base generation approach. The latter captures inbreeding from pedigree relationships up to the new base generation tracking (*t* generations back) with the assumption that base population animals are unrelated.

#### Pre-defined number of generations or segment length approach

When pedigree information is used, a fixed arbitrary number of generations is used as a threshold to cluster inbreeding into old and new classes. Silió et al. [40] and Ferenčaković et al. [41] used five generations to calculate F_new _ 5 _ Lit_ and F_old _ 5 _ Lit_. Saura et al. [23] and Clark et al. [13] used a fixed 5 Mb threshold to classify old (F_short _ 5 _ Lit_) and new (F_long _ 5 _ Lit_) inbreeding. Pemberton et al. [46] used a three-component mixture Gaussian model as implemented in *mclust (v.5)* R package [65] to classify ROH segments into short, medium and long classes. In the current study, only two components (short and long) were considered due to limitations in the genomic data with corresponding inbreeding coefficients F_short _ Mclust_ and F_long _ Mclust_. The calculation of the individual pedigree (new vs. old) and ROH (short vs. long) based inbreeding coefficients is as presented in eqs. 1, 3, and 4.

### Statistical analysis of inbreeding depression

Univariate models were used for each trait separately to assess inbreeding depression by regressing the trait phenotypes (BW, WW, YW, and ADG) on inbreeding coefficients:

5 $$ \mathbf{y}=\mathbf{Xb}+\mathbf{Zu}+\mathbf{Wm}+\mathbf{Sp}+\mathbf{e}, $$

where **y** is a vector of phenotypes for the trait of interest, **b** is the vector of fixed effects of sex, birth year, age (as covariate for WW and YW) and regressions on inbreeding coefficients, **u** is a vector of random direct genetic effects, **m** and **p** are the vectors of random maternal genetic and maternal permanent environmental effects (only for BW and WW), and **e** is the vector of random residuals. The incidence matrices **X,Z,W**, and **S** relate the records to fixed, direct genetic, maternal genetic, and maternal permanent environmental effects, respectively. For each approach, the new and old inbreeding coefficients were fit simultaneously in the model in order to account for potential correlations between them. Inbreeding effects were estimated using the BLUPF90 family programs [88]. The effect of inbreeding on a given trait was assessed based on the significance of its associated regression coefficient using a *t*-statistic unit ($\left|\frac{\hat{\upbeta}}{\mathrm{SE}}\right|>2$). A detailed description of the implementation steps of the proposed algorithm to determine the threshold to discriminate between recent and old inbreeding is provided in Appendix 2.

The comparison between the different approaches was based on adjusted R-squared (Adj.R2), root mean squared error (RMSE), Akaike’s information criteria (AIC), and Bayesian information criteria (BIC). Only fixed effects were included in the models for assessing inbreeding depression. The modelr [89] and stats [86] packages of R were used for the implementation of model comparisons.

## Supplementary Information

**Additional file 1: Figure S1.** Distributions of pedigree-based inbreeding (F_ped_ in % ) for different number of generations between an animal and its earliest ancestors (MaxGen).

**Additional file 2: Table S1.** Distributions of pedigree-based inbreeding (F_ped_ in % ) for different discrete equivalent complete generations (ECG).

**Additional file 3: Table S2.** Distribution of new and old pedigree-based inbreeding estimates using the proposed and existing approaches (genotyped animals only; *n* = 785).

**Additional file 4: Table S3.** Distribution of new (long ROH) and old (short ROH) inbreeding using the proposed and existing ROH-based approaches (genotyped animals; *n* = 785).

**Additional file 5: Table S4.** Distribution of runs of homozygosity segments and their associated inbreeding (*n* = 785).

**Additional file 6: Table S5.** Number of ROH segments at different interval lengths (*n* = 785).

**Additional file 7: Appendix 1.** Example source code for the estimation of ROH-based inbreeding.

**Additional file 8: Appendix 2.** A detailed description of the implementation steps of the algorithm.

## Data Availability

All the necessary information needed to support the results of this paper are included within the article and its additional files. The data supporting the findings of this study are available upon request from the corresponding author with permission from the USDA Agricultural Research Service (El Hamidi Hay: elhamidi.hay@usda.gov). Additional supporting figures, tables, and Appendices are included as Additional files.

## Abbreviations

ADG: Average daily gain between weaning and yearling
Adj.R2: Adjusted R-squared
AIC: Akaike’s Information Criteria
$\hat{\upbeta}$: the estimated regression coefficient
BIC: Bayesian information criteria
BW: Birth weight
ECG: Equivalent complete generation
F: Inbreeding coefficient
F_anc _ Kal_: Ancestral inbreeding component based on Kalinowski et al. (2000)‘s method
F_long _ 5 _ Lit_: Inbreeding coefficient due to long ROH based on 5 Mb as defined from the literature
F_long _ m_: Inbreeding coefficient due to long ROH (≥ m Mb) class
F_long _ Mclust_: Inbreeding coefficient due to long ROH based on model-based clustering method;
F_new _ 5 _ Lit_: New inbreeding coefficient based on 5 generations for recent generations according to the literature
F_new _ Kal_: New inbreeding component based on Kalinowski et al. (2000)‘s method
F_new _ t_: New inbreeding coefficients estimated from the first t generations back in the pedigree
F_old _ 5 _ Lit_: Old inbreeding coefficient based on 5 generations for recent generations according to the literature
F_old _ t_: Old inbreeding coefficients estimated from the first *t* generations back in the pedigree
F_ped_: Pedigree-based inbreeding coefficient
F_ROH_: ROH-based inbreeding coefficient
F_short _ 5 _ Lit_: Inbreeding coefficient due to short ROH based on 5 Mb as defined from the literature
F_short _ m_: Inbreeding coefficient due to short ROH (< m Mb) class
F_short _ Mclust_: Inbreeding coefficient due to short ROH based on model-based clustering method;
HBD: Homozygous by descent
IBD: Identical by descent
Kalinowski: Classification of inbreeding based on Kalinowski et al. (2000)
MaxGen: The maximum number of generation traced back
Mb: Megabase
P_5L: Classification of inbreeding based on 5 generations according to literature
P_10P: Classification of inbreeding based on 10 generations determined by the proposed method
P_11P: Classification of inbreeding based on 11 generations determined by the proposed method
RMSE: Root Mean Squared Error
ROH: Runs of homozygosity
ROH_5L: Classification of inbreeding based on 5 Mb according to literature
ROH_7P: Classification of inbreeding based on 7 Mb determined by the proposed method
ROH_9P: Classification of inbreeding based on 9 Mb determined by the proposed method
ROH_13P: Classification of inbreeding based on 13 Mb determined by the proposed method
ROH_Mclust: Classification of inbreeding based on model-based clustering method
SE: Standard error
SNP: Single nucleotide polymorphism
WW: Weaning weight
YW: Yearling weight
