# Supplementary material for: Grid search approach to discriminate between old and recent inbreeding using phenotypic, pedigree and genomic information
Source: BMC Genomics. 2021 Jul 13;22:538. doi: 10.1186/s12864-021-07872-z (PMC8278650; doi:10.1186/s12864-021-07872-z)
Supplement: Supplementary file 1 — Additional file 1: Figure S1. Distributions of pedigree-based inbreeding (Fped in % ) for different number of generations between an animal and its earliest ancestors (MaxGen). [file 12864_2021_7872_MOESM1_ESM.docx]

**
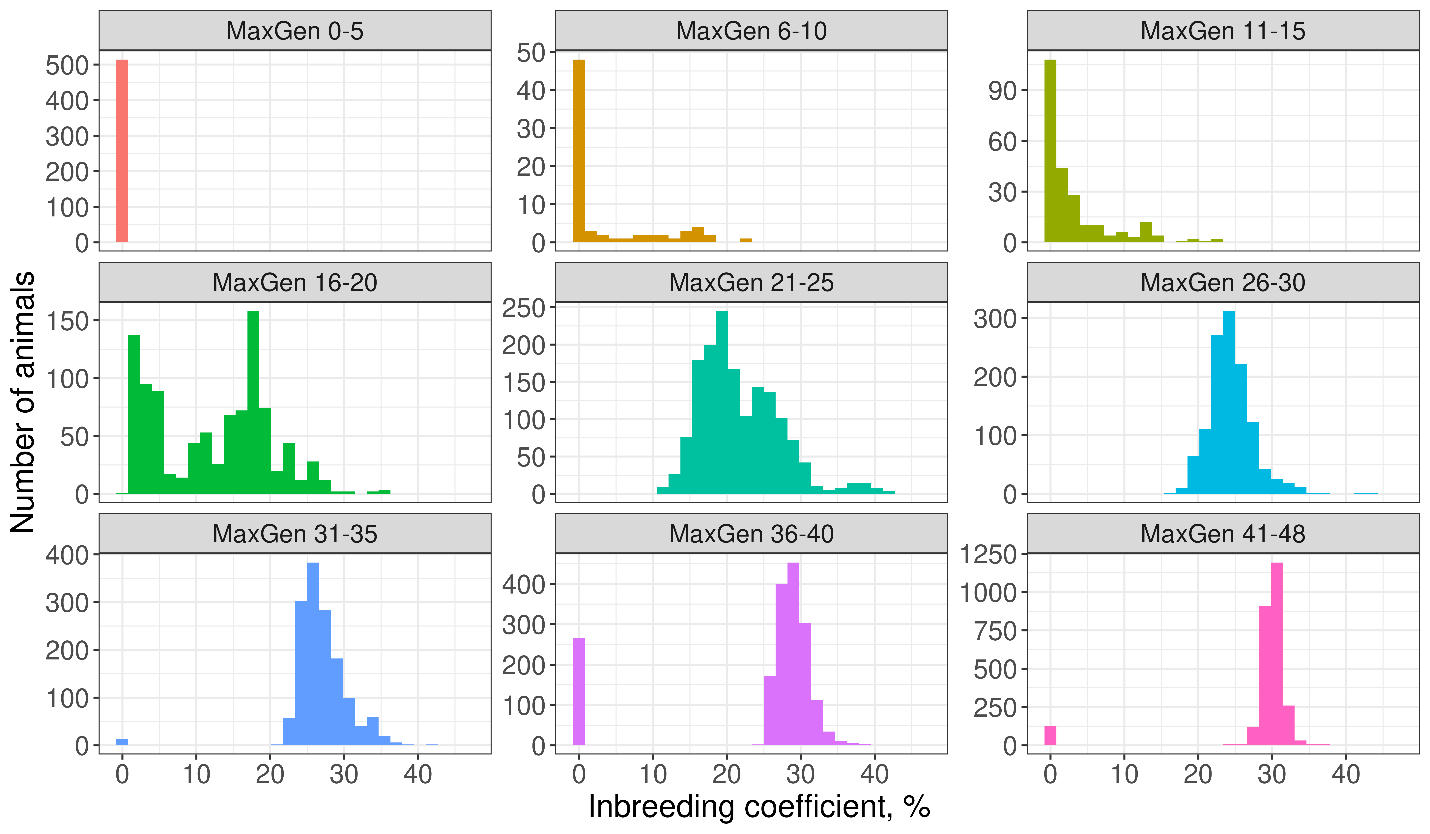
**

**Fig. S1** Distributions of pedigree-based inbreeding ($F_{\mathrm{ped}} in \%)$ for different number of generations between an animal and its earliest ancestors (MaxGen).
