# Supplementary material for: Grid search approach to discriminate between old and recent inbreeding using phenotypic, pedigree and genomic information
Source: BMC Genomics. 2021 Jul 13;22:538. doi: 10.1186/s12864-021-07872-z (PMC8278650; doi:10.1186/s12864-021-07872-z)
Supplement: Supplementary file 2 — Additional file 2: Table S1. Distributions of pedigree-based inbreeding (Fped in % ) for different discrete equivalent complete generations (ECG). [file 12864_2021_7872_MOESM2_ESM.docx]

**Table S1** Distributions of pedigree-based inbreeding ($F_{\mathrm{ped}} in \%)$ for different discrete equivalent complete generations (ECG)

| **ECG** | **All animals (n = 10478)** | | | |  | **Genotyped animals (n = 785)** | | | |
| --- | --- | --- | --- | --- | --- | --- | --- | --- | --- |
|  | **n** | **Mean (SD)** | **Min** | **Max** |  | **n** | **Mean (SD)** | **Min** | **Max** |
| 0-5 | 717 | 0.45 (1.96) | 0.00 | 16.02 |  | 0 | - | - | - |
| 6-10 | 1268 | 12.25 (7.92) | 0.00 | 37.06 |  | 1 | 0.00 (-) | - | - |
| 11-15 | 2509 | 19.34 (9.25) | 0.00 | 43.38 |  | 26 | 5.27 (9.87) | 0.00 | 26.68 |
| 16-20 | 2161 | 26.85 (3.00) | 13.17 | 46.35 |  | 49 | 25.79 (2.19) | 22.43 | 31.86 |
| 21-25 | 2882 | 29.47 (1.77) | 25.56 | 46.74 |  | 267 | 29.27 (1.19) | 25.98 | 33.66 |
| 26-28 | 941 | 30.78 (1.21) | 28.78 | 39.98 |  | 442 | 31.06 (1.34) | 28.95 | 39.98 |
