# Supplementary material for: Grid search approach to discriminate between old and recent inbreeding using phenotypic, pedigree and genomic information
Source: BMC Genomics. 2021 Jul 13;22:538. doi: 10.1186/s12864-021-07872-z (PMC8278650; doi:10.1186/s12864-021-07872-z)
Supplement: Supplementary file 3 — Additional file 3: Table S2. Distribution of new and old pedigree-based inbreeding estimates using the proposed and existing approaches (genotyped animals only; n = 785). [file 12864_2021_7872_MOESM3_ESM.docx]

**Table S2** Distribution of new and old pedigree-based inbreeding estimates using the proposed and existing approaches (genotyped animals only; n = 785)

| **Inbreeding ^a^** | | **Mean** | **SD** | **Min** | **Max** |
| --- | --- | --- | --- | --- | --- |
| Total | $F_{\mathrm{ped}}$ | 0.2923 | 0.0526 | 0 | 0.3998 |
| Existing | $F_{new\_5\_Lit}$ | 0.0207 | 0.0221 | 0 | 0.2285 |
|  | $F_{old\_5\_Lit}$ | 0.2716 | 0.0520 | 0 | 0.3019 |
|  | $F_{new\_Kal}$ | 0.0279 | 0.0091 | 0 | 0.1036 |
|  | $F_{anc\_Kal}$ | 0.1182 | 0.0311 | 0 | 0.2210 |
| Proposed | $F_{new\_10}$ | 0.1044 | 0.0414 | 0 | 0.2764 |
|  | $F_{old\_10}$ | 0.1879 | 0.0562 | 0 | 0.2532 |
|  | $F_{new\_11}$ | 0.1312 | 0.0486 | 0 | 0.2896 |
|  | $F_{old\_11}$ | 0.1611 | 0.0600 | 0 | 0.2352 |

^a^ Total = pedigree inbreeding ($F_{\mathrm{ped}}$) using all animals; Existing = old and new inbreeding using existing approaches; Proposed = old and new inbreeding using the proposed approach; $F_{new\_5\_Lit}$ and $F_{old\_5\_Lit}$ = new and old inbreeding based on 5 generation threshold; $F_{new\_Kal}$, and $F_{anc\_Kal}$ = new and ancestral inbreeding based on Kalinowski et al. (2000); $F_{new\_t}$, and $F_{old\_t}$ = new and old inbreeding based on tracing the pedigree back to $t$ ancestral generations ($t$ = 10 or 11)
