# Supplementary material for: Grid search approach to discriminate between old and recent inbreeding using phenotypic, pedigree and genomic information
Source: BMC Genomics. 2021 Jul 13;22:538. doi: 10.1186/s12864-021-07872-z (PMC8278650; doi:10.1186/s12864-021-07872-z)
Supplement: Supplementary file 4 — Additional file 4: Table S3. Distribution of new (long ROH) and old (short ROH) inbreeding using the proposed and existing ROH-based approaches (genotyped animals; n = 785). [file 12864_2021_7872_MOESM4_ESM.docx]

**Table S3** Distribution of new (long ROH) and old (short ROH) inbreeding using the proposed and existing ROH-based approaches (genotyped animals; n = 785)

| **Inbreeding ^a^** | | **Mean** | **SD** | **Min** | **Max** |
| --- | --- | --- | --- | --- | --- |
| Total | $F_{\mathrm{ROH}}$ | 0.2430 | 0.0539 | 0.0103 | 0.4085 |
| Existing | $F_{long\_5\_Lit}$ | 0.1785 | 0.0448 | 0 | 0.3332 |
|  | $F_{short\_5\_Lit}$ | 0.0645 | 0.0139 | 0.0077 | 0.1054 |
|  | $F_{long\_Mclust}$ | 0.0918 | 0.0310 | 0 | 0.2068 |
|  | $F_{short\_Mclust}$ | 0.1512 | 0.0323 | 0.0103 | 0.2241 |
| Proposed | $F_{long\_7}$ | 0.1278 | 0.0371 | 0 | 0.2702 |
|  | $F_{short\_7}$ | 0.1152 | 0.0245 | 0.0103 | 0.1656 |
|  | $F_{long\_9}$ | 0.0921 | 0.0310 | 0 | 0.2068 |
|  | $F_{short\_9}$ | 0.1509 | 0.0322 | 0.0103 | 0.2230 |
|  | $F_{long\_13}$ | 0.0505 | 0.0229 | 0 | 0.1409 |
|  | $F_{short\_13}$ | 0.1925 | 0.0417 | 0.0103 | 0.2863 |

^a^ Total = inbreeding based on all ROH segments ($F_{\mathrm{ROH}}$); Existing = old and new inbreeding using existing approaches; Proposed = old and new inbreeding using the proposed approach; $F_{long\_Mclust}{and F}_{short\_Mclust}$ = long and short ROH segment inbreeding using the model-based clustering method; $F_{long\_5\_Lit}$ and $F_{short\_5\_Lit}$ = long and short ROH segment inbreeding based on 5 Mb threshold; $F_{long\_m}$ and $F_{short\_m}$ = long and short ROH segment inbreeding based on the proposed method ($m$ = 7, 9, or 13 Mb).
