# Supplementary material for: Grid search approach to discriminate between old and recent inbreeding using phenotypic, pedigree and genomic information
Source: BMC Genomics. 2021 Jul 13;22:538. doi: 10.1186/s12864-021-07872-z (PMC8278650; doi:10.1186/s12864-021-07872-z)
Supplement: Supplementary file 5 — Additional file 5: Table S4. Distribution of runs of homozygosity segments and their associated inbreeding (n = 785). [file 12864_2021_7872_MOESM5_ESM.docx]

**Table S4** Distribution of runs of homozygosity segments and their associated inbreeding (n = 785)

| **Classification ^a^** | **ROH class** | **Number of ROH** | | | **Total length (Mb)** | | | **Inbreeding coefficients (%)** | | |
| --- | --- | --- | --- | --- | --- | --- | --- | --- | --- | --- |
|  |  | Mean (SD) | Min | Max | Mean (SD) | Min | Max | Mean (SD) | Min | Max |
| All ROH |  | 100.6 (20.8) | 7 | 146 | 610.5 (135.5) | 25.9 | 1026.4 | 24.30 (5.39) | 1.03 | 40.86 |
| ROH_Mclust | Short | 83.9 (17.6) | 7 | 125 | 379.8 (81.1) | 25.9 | 563.1 | 15.12 (3.23) | 1.03 | 22.41 |
|  | Long | 16.7 (5.4) | 0 | 35 | 230.7 (77.8) | 0 | 519.5 | 9.18 (3.10) | 0 | 20.68 |
| ROH_5L | Short | 50.9 (11.2) | 6 | 78 | 162.1 (34.9) | 19.3 | 264.9 | 6.45 (1.39) | 0.77 | 10.54 |
|  | Long | 49.7 (11.7) | 0 | 82 | 448.4 (112.6) | 0 | 837.0 | 17.85 (4.48) | 0 | 33.32 |
| ROH_7P | Short | 72.5 (15.4) | 7 | 106 | 289.5 (61.6) | 25.9 | 416.0 | 11.52 (2.45) | 1.03 | 16.56 |
|  | Long | 28.1 (7.7) | 0 | 55 | 321.0 (93.3) | 0 | 678.9 | 12.78 (3.71) | 0 | 27.02 |
| ROH_9P | Short | 83.8 (17.6) | 7 | 124 | 379.0 (81.0) | 25.9 | 560.1 | 15.09 (3.22) | 1.03 | 22.30 |
|  | Long | 16.8 (5.4) | 0 | 35 | 231.5 (77.9) | 0 | 519.5 | 9.21 (3.10) | 0 | 20.68 |
| ROH_13P | Short | 93.6 (19.6) | 7 | 137 | 483.5 (104.7) | 25.9 | 719.2 | 19.25 (4.17) | 1.031 | 28.63 |
|  | Long | 6.9 (3.0) | 0 | 18 | 127.0 (57.4) | 0 | 354.0 | 5.06 (2.29) | 0 | 14.09 |

^a^ All ROH = all runs of homozygosity (ROH) segments; ROH_Mclust = classification of inbreeding based on model-based clustering method; ROH_5L = classification of inbreeding based on 5 Mb according to literature; ROH_7P, ROH_9P, and ROH_13P = classification of inbreeding based on 7, 9, and 13 Mb, respectively, as determined by the proposed method
