# Supplementary material for: Grid search approach to discriminate between old and recent inbreeding using phenotypic, pedigree and genomic information
Source: BMC Genomics. 2021 Jul 13;22:538. doi: 10.1186/s12864-021-07872-z (PMC8278650; doi:10.1186/s12864-021-07872-z)
Supplement: Supplementary file 6 — Additional file 6: Table S5. Number of ROH segments at different interval lengths (n = 785). [file 12864_2021_7872_MOESM6_ESM.docx]

**Table S5** Number of ROH segments at different interval lengths ^a^ (n = 785)

| **Interval (Mb) ^b^** | **Total number of ROH ^c^** | **Number of ROH per animal** | | | |
| --- | --- | --- | --- | --- | --- |
|  |  | **Mean** | **SD** | **Min** | **Max** |
| 0.1 – 0.2 | 53 | 0.07 | 0.26 | 0 | 2 |
| 0.2 – 0.3 | 1024 | 1.30 | 0.97 | 0 | 4 |
| 0.3 – 0.4 | 767 | 0.98 | 0.91 | 0 | 5 |
| 0.4 – 0.5 | 1224 | 1.56 | 1.17 | 0 | 7 |
| 0.5 – 0.6 | 863 | 1.10 | 1.07 | 0 | 7 |
| 0.6 – 0.7 | 800 | 1.02 | 1.02 | 0 | 5 |
| 0.7 – 0.8 | 1268 | 1.62 | 1.15 | 0 | 6 |
| 0.8 – 0.9 | 1045 | 1.33 | 1.16 | 0 | 5 |
| 0.9 – 1.0 | 1195 | 1.52 | 1.19 | 0 | 7 |

^a^ parameter’s settings: minimum length of 100 kb, minimum of 5 SNP per ROH call

^b^ ROH segments length

^c^ Total number of ROH segments across all animals
