# Supplementary material for: Grid search approach to discriminate between old and recent inbreeding using phenotypic, pedigree and genomic information
Source: BMC Genomics. 2021 Jul 13;22:538. doi: 10.1186/s12864-021-07872-z (PMC8278650; doi:10.1186/s12864-021-07872-z)
Supplement: Supplementary file 8 — Additional file 8: Appendix 2. A detailed description of the implementation steps of the algorithm. [file 12864_2021_7872_MOESM8_ESM.docx]

**Appendix 2: A detailed description of the implementation steps of the algorithm**

**Step 1. Calculation of age-specific inbreeding coefficients**

Heuristically predefined thresholds were used for the classification of inbreeding into new and old classes. Specifically, the threshold based on number of generations, $t_{i}$, was varied between 3 and 16 and the threshold based on the ROH segment length, $m_{i}$ varied between 3 and 17 Mb.

Let $\boldsymbol{t}\boldsymbol{=}(3,4,5,\ldots,16)'$ and $\boldsymbol{m}\boldsymbol{=}(3,5,7,\ldots,17)'$ be the vectors of predefined thresholds for pedigree and ROH segment length-based classification of inbreeding, respectively.

For each threshold (based on number of generations or ROH length), new and old inbreeding were computed.

**Step 2. Creating a matrix to store results**

For each scenario (using pedigree or ROH segments), an empty matrix, ***Sol***, was created to store the results (effects of age specific inbreeding and their associated standard errors). The matrix **Sol** has 6 columns and 14 (pedigree) or 8 (ROH segments) rows. Each row of the matrix includes the sequential index for the threshold, the threshold value, estimate of the regression for new inbreeding and its associated standard error (SE), and estimate of the regression for old inbreeding and its associated SE.

**Step 3. Solving for inbreeding depression**

For each threshold *i*, the estimates for the regression coefficients of age-specific inbreeding and their associated SE are set to zero:

$$\hat{\beta}_{new}^{i}=0$$

$${SE}_{new}^{i}=0$$

$\hat{\beta}_{old}^{i}=0$

$${SE}_{old}^{i}=0$$

Two columns containing the age-specific inbreeding coefficients ($F_{new\_t}$ and $F_{old\_t}$), calculated as indicated in step 1, are added to the data file that already contains all information necessary for solving the inbreeding depression model. $F_{new\_t}$ and $F_{old\_t}$ are standardized to have zero mean and variance equal to 1 (Z-scores).

Using the specified linear model, the estimates of the effects of age-specific inbreeding and their associated SE are obtained for threshold *i*:

$$\hat{\beta}_{new}^{i}=\hat{\beta}_{new\_t}$$

$${SE}_{new}^{i}={SE}_{new\_t}$$

$$\hat{\beta}_{old}^{i}=\hat{\beta}_{old\_t}$$

$${SE}_{old}^{i}={SE}_{old\_t}$$

and the element of the matrix **Sol** are updated:

$\mathbf{Sol}\left[ i,1 \right]=i$ # index i

$\mathbf{Sol}\left[ i,2 \right]=t$ # threshold t

$\boldsymbol{Sol}\left[ i,3 \right]=\hat{\beta}_{new}^{i}$ # effect of new inbreeding

$\boldsymbol{Sol}\left[ i,4 \right]={SE}_{new}^{i}$ # SE associated with the effect of new inbreeding

$\boldsymbol{Sol}\left[ i,5 \right]=\hat{\beta}_{old}^{i}$ # effect of old inbreeding

$\boldsymbol{Sol}\left[ i,6 \right]={SE}_{old}^{i}$ # SE associated with the effect of old inbreeding

The process will be repeated for all values of $i=(1,2,3,\ldots,n)$ with n=14 (pedigree) or 8 (ROH segments).

**Step 4. Determination of the optimum threshold**

The optimum threshold for the classification of inbreeding into new and old classes is identified using the following equations

$\hat{t}={Min}_{t}\left( \hat{\beta}_{new\_t}<\hat{\beta}_{old\_t} \right)$ for pedigree

$\hat{m}={Max}_{m}\left( \hat{\beta}_{long\_m}<\hat{\beta}_{short\_m} \right)$ for ROH segments

Step 3 is trait specific and it has to be repeated for each trait.
